# A Novel Diagnostic Imaging Method for the Early Detection of Pancreatic Cancer

**DOI:** 10.3390/diagnostics13122080

**Published:** 2023-06-15

**Authors:** Masataka Kikuyama

**Affiliations:** Department of Gastroenterology, Tokyo Women’s Medical University, 8-1 Shinjuku-ku, Tokyo 162-8666, Japan; kikuyama.masataka@twmu.ac.jp

Pancreatic ductal adenocarcinoma (PDAC) has an extremely poor prognosis, with a survival rate of less than 10% [1,2]. Various factors have been associated with this phenomenon. For example, effective chemotherapy remains under development [3], and patients referred for surgical intervention must be selected using strict criteria [4]. However, the most profound reason for the poor prognosis is the limited early-stage detection of PDAC [5]. To improve the prognosis, an early-stage diagnosis of PDAC is essential. Therefore, the selection of an effective diagnostic method is critical.

Ultrasonography (US), computed tomography (CT), and magnetic resonance imaging (MRI) are the commonly used methods for PDAC detection (Table 1). US is easy to perform at the bedside and is noninvasive. CT aids in diagnosing malignant tumors of the liver, kidney, adrenal gland, and lymph nodes [6]; moreover, it provides information in emergencies [7]. MRI is essential for the qualitative diagnosis of a tumor in the abdominal organs [6]; in particular, diffusion-weighted MRI can identify an abnormal lesion in the body that cannot be identified using CT [8].

US has limited usage in observing the entire abdominal cavity and diagnosing abnormal findings in a clinical setting, including at the scene of an emergency [9]. Moreover, the diagnostic quality of US depends on the operators [10]. The pancreas is a retroperitoneal organ behind the stomach that is difficult to observe using US [11]. Therefore, US has limited efficacy in diagnosing pancreatic diseases. In fact, US’s sensitivity in diagnosing PDAC is reported to be as low as 69.01% [12]. At an early stage, US has a tumor detection rate of only 52.6% [13].

CT and MRI are also limited in diagnosing PDAC [14,15], although these methods provide a clear image of the abdominal organ. CT is not sufficient to diagnose tumors < 20 mm [14]. MRI is good for depicting a cystic lesion but inefficient in diagnosing a small, solid lesion [15]. Diffusion-weighted MRI is sufficiently efficient in diagnosing PDAC, but no studies have revealed its ability to detect small PDACs [16]. Positron emission tomography (PET) has been used to identify abnormal abdominal lesions, including cancer [17]. However, it is not superior to prior diagnostic methods for PDAC [18].

In other words, any conventional diagnostic method has limitations in diagnosing PDAC at the early stage.

Recently, endoscopic ultrasonography (EUS) has been reported as a diagnostic tool for detecting early-stage PDAC [19,20,21,22] with a prominent negative predictive value [19]. Essentially, US has a superior ability to reveal minute abnormalities in organs [23]; however, the circumstances differ in the pancreas. As described above, this organ is retroperitoneal, and US cannot detect minute pancreatic lesions pancreas. Moreover, due to its position behind the stomach, imaging is obstructed by air in the stomach [11], limiting its ability to observe the entire pancreas.

The ultrasound probe must be placed close to the pancreas, or an endoscope can be used to overcome these problems. The endoscope tip easily reaches the stomach and duodenum, and the pancreas can be observed precisely using US through the walls of these organs. Several reports have described that EUS is superior to other diagnostic methods, such as CT, MRI, US, and PET, in identifying PDACs, especially small ones [20,21].

In addition to its ability to detect small PDACs, EUS provides an opportunity to infer the histopathological characteristics of pancreatic lesions [24]. That is, using the technique of EUS-guided fine-needle aspiration (EUS-FNA), cytological or histopathological diagnosis can be achieved with high sensitivity and specificity for a pancreatic tumor [24,25,26]. Using EUS-FNA, we were able to diagnose and treat pancreatic diseases accurately. Moreover, various ideas have been reported for a more accurate diagnosis using EUS-FNA, such as the development of new puncture needles [27,28] and novel methods to aspirate the tissue [29,30]. Contrast-enhanced (CE) EUS [31] and elastography [32] have recently been used to observe pancreatic lesions. These data provide information for estimating the histopathological characteristics of pancreatic lesions. However, the information obtained from these examinations requires objectivity, and further estimation is necessary.

Compared with conventional methods, including CT and MRI, EUS has the potential to diagnose small PDACs. However, even if EUS can diagnose a small PDAC, the tumor is invasive and can potentially metastasize because PDAC originates from the pancreatic ductal epithelium, invades the surrounding parenchyma, and forms a tumor that can be recognized. The five-year survival rate is unsatisfactory despite the small size of Ia and Ib tumors (80.4 and 50.0%, respectively) [5]. A case of small PDACs with a diameter of 4 mm and metastasis to the lymph nodes around the pancreas has been reported [33].

High-grade pancreatic intraepithelial neoplasia/carcinoma in situ (HGP/CIS) deserves the definition of “early pancreatic cancer” because it does not metastasize. However, direct observation of carcinoma in situ is difficult even when using an endoscopic method because of the small diameter of the main pancreatic duct, and we have no information on the macroscopic epithelial findings of HGP/CIS. In other words, diagnosing HGP/CIS using direct observation is impossible.

However, if we recognize secondary signs suggestive of HGP/CIS, HGP/CIS can be diagnosed. Main pancreatic duct (MPD) stricture has been accepted as a secondary sign of early-stage PDAC, including HGP/CIS [13,34]. More sensitive and specific secondary symptoms of HGP/CIS have been recently explored, including focal pancreatic parenchymal atrophy (FPPA) [35,36]. HGP/CIS influences the surrounding pancreatic parenchyma through a mechanism that causes atrophy, and this change can be recognized on CT or MRI [36,37]. Moreover, this change occurs 38 months [37] or at least 1 year before the diagnosis of PDAC [38].

Moreover, the cytological examination of pancreatic juice has advanced. Originally, pancreatic juice cytology had high sensitivity for diagnosing PDAC, but the results were not always satisfactory owing to its low sensitivity [39]. However, a novel cytologic method, serial pancreatic juice aspiration cytologic examination (SPACE) [40,41], has dramatically improved the diagnosis of PDAC by means of pancreatic juice cytology.

FPPA is a secondary finding suggesting HGP/CIS that can be recognized on MRI or CT [36,37,38,41,42]. FPPA presents as an irregularly shaped focal defect in the pancreatic parenchyma, which can be recognized as an indentation of the parenchyma on CT or MRI (Figure 1) [35,36] showing asymmetric changes concerning the MPD. According to the shape, FPPA is classified into three types: cave-in, slimness, and slit [35]. The cave-in type presents as a focal indentation of the pancreatic surface; the slimness type as longitudinal but focal atrophy of the pancreatic parenchyma with a shaggy appearance; and the slit type as a cuneiform defect of the pancreatic parenchyma.

Histopathologically, the FPPA area comprises replaced adipose tissue, sometimes containing fibrotic and chronic inflammatory cells (Figure 2) [36]. The margin of the area is sharp and clear, and the surrounding pancreatic parenchyma shows no significant changes, such as inflammatory cell infiltration, fibrosis, or atrophy. FPPA is observed around or adjacent to the pancreatic duct, with neoplastic changes in the pancreatic ductal epithelium.

MPD strictures are reliable signs of PDAC, including HGP/CIS [13,34]. However, FPPA has been reported to be more sensitive and helpful in identifying HGP/CIS of the pancreatic duct than MPD strictures [35]. Among patients with FPPA, 46% had a positive result (adenocarcinoma and suspicious) on SPACE, and 65% of those who underwent surgery due to the result had a final histopathological diagnosis of HGP/CIS [36].

FPPA could be an alternative secondary sign of early-stage PDAC, including HGP/CIS. To evaluate whether FPPA is associated with PDAC development, Nakahodo et al. reported FPPA was identified in 47/170 (28%) patients before PDAC diagnosis [37], and the median duration from FPPA detection to diagnosis was 35 months [37]. Moreover, the resolution of the FPPA was a sign of PDAC development because PDAC developed and filled the atrophied pancreatic parenchyma [37]. Thus, identifying FPPA provides an opportunity to diagnose and treat early-stage PDAC.

However, any degree of neoplastic change in the pancreatic duct could induce FPPA, although FPPA occurs in association with HGP/CIS because patients undergoing surgery due to positive SPACE results do not always have histopathological results of HGP/CIS [37]. FPPA has also been observed around low-grade pancreatic intraepithelial neoplasia (LGP). Thus, we were concerned about the difference between FPPA in HGP/CIS and LGP.

FPPA was studied by focusing on the size of the area of FPPA [42] and it was revealed that a larger area of FPPA (>269.79 mm^2^, *p* = 0.0001) was related to positive results (adenocarcinoma and suspicion) of pancreatic juice cytology on SPACE. Moreover, FPPA commonly accompanies a cystic lesion, and when the cystic lesion is prominent and clinically diagnosed as intraductal papillary mucinous neoplasia, the rate of positive pancreatic juice cytology results on SPACE is significantly higher [42]. Surgery based on positive results can lead to a histopathological diagnosis of HGP/CIS [42].

Although FPPA is closely related to neoplastic changes in the pancreatic duct, including HGP/CIS, the mechanism of FPPA has not been identified, and various conjectures have been proposed, such as pancreatic parenchymal cell apoptosis caused by focal acute pancreatitis [43] and acinar ductal metaplasia [44]. Acute pancreatitis could be induced due to pancreatic juice obstruction by the mucin produced by the neoplastic cells, while histopathological examination of the resected specimen revealed that mucin production is not always prominent in branch duct obstruction. Acinar ductal metaplasia leads to atrophy; however, fat replacement is not accompanied by this phenomenon. Further investigation is required to clarify the mechanism of action of FPPA.

FPPA can be observed on EUS as a blurred hypoechoic area (Figure 3) [22,45]. The pancreatic parenchyma is hyperechoic. If the parenchyma is atrophied and the area is replaced by fibrosis and fat, echogenicity is lost, and the area becomes hypoechoic. However, this area is not clearly demarcated and can be blurred. In some cases, fibrosis related to HGP/CIS strictures the MPD and shows an MPD stricture surrounded by hypoecho, which is known as a hypoechoic stricture [22].

A newly developed cytologic examination of pancreatic juice for PDAC, namely SPACE, has a high sensitivity for PDAC, especially for HGP/CIS [40,41]. Pancreatic juice cytology was repeated at least six times by placing a nasopancreatic duct drainage tube (Figure 4). The pancreatic juice for cytology was obtained using endoscopic retrograde cholangiopancreatography; placing a nasopancreatic drainage tube on SPACE could improve sensitivity because it allowed repeated gain of pancreatic juice and made it possible to perform cytology multiple times; this improved sensitivity for PDAC [40]. If HGP/CIS is suspected based on the recognition of FPPA, SPACE can contribute to the diagnosis. Thus, SPACE can improve the prognosis of PDAC.

However, SPACE may cause post-endoscopic cholangiopancreatography pancreatitis (PEP) [36,40]. In addition to the obstruction of pancreatic juice flow from the pancreatic branch duct during a nasopancreatic drainage tube placement, pancreatic duct injury during the introduction of the guidewire into the pancreatic duct could induce PEP. However, although hyperamylasemia is relatively common [36], the ratio of PEP is not always high, and severe PEP is rare [36,40]. As pancreatic juice drainage using a nasopancreatic drainage tube can be used to treat acute pancreatitis [46], the incidence of PEP during SPACE is low. Another problem with SPACE is that it impels the patient to stay in the hospital for several days because of the maintenance of the placed nasopancreatic tube, although meals are not restricted.

Therefore, SPACE allows early-stage diagnosis of PDAC; however, careful selection of candidates that require SPACE should be performed because of its inherent complications and burden.

High-risk factors for PDAC, such as a familial history of PDAC, pancreatic cystic lesions, and elderly-onset diabetes mellitus, are well known [47]. Patients at risk of PDAC are candidates for pancreatic examination. The strategy considered efficient for diagnosing early-stage PDAC is shown in Figure 5.

EUS is the first-choice replacement for MRI or CT because EUS is the only examination able to reveal a small PDAC. When a tumor is recognized, EUS-FNA is selected to confirm the diagnosis. When no tumor is recognized despite a high risk of being present, examination for PDAC should be repeated. The time interval between examinations is important.

The mean tumor volume doubling time (TVDT) of PDAC is approximately five months (21.2–255 days) [48,49]. For example, a PDAC with a diameter of 4 mm, which is difficult to recognize using any diagnostic method, including EUS [33], grows into a tumor of 5.2 mm within six months, which can be recognized only with EUS. If EUS is used to observe patients at risk of PDAC, a six-month interval is considered reasonable.

However, some PDACs have shorter TVDTs. If the TVDT is 30 days, a tumor of 4 mm grows to 15 mm within six months, classified as TS1c, with an unfavorable prognosis and high potential for metastasis. Once a tumor becomes invasive, its prognosis depends on its characteristics, and the diagnosis of small PDACs depends on the timing of the examination. Diagnosing PDAC at stage 0 HGP/CIS before it becomes invasive is ideal.

Before a recognizable tumor emerges, FPPA develops due to neoplastic changes in the pancreatic duct, which can be recognized on MRI or CT. When EUS does not reveal a tumor but FPPA is identified, neoplastic changes in the pancreatic duct are suspected, and SPACE can be used for diagnosing HGP/CIS.

However, if repeated EUS-FNAs cannot diagnose PDAC despite recognition of a tumor, an alternative method in the form of SPACE could be selected because PDAC originates from the pancreatic duct epithelium and SPACE, which can obtain epithelial cells, is more sensitive than EUS-FNA for diagnosing PDAC [22]. Conversion of the examination results from EUS-FNA to SPACE should also be considered.

In conclusion, the conventional methods for diagnosing PDAC are ineffective in improving prognosis. The early diagnosis and treatment of HGP/CIS are ideal and have become possible. Furthermore, identifying FPPA is essential.

## Figures and Tables

**Figure 1:**
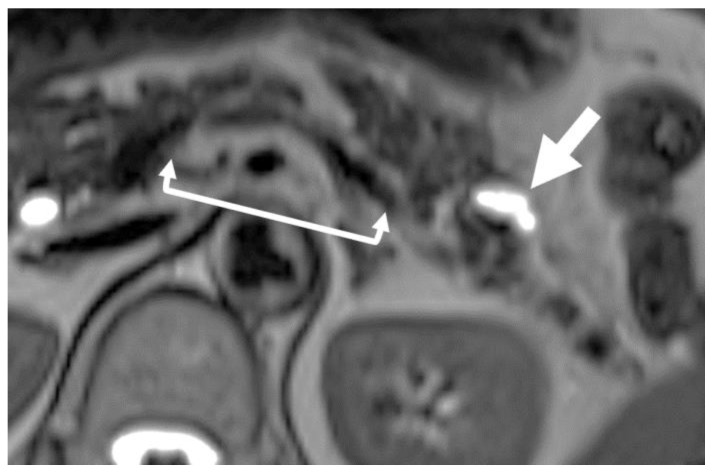
Focal pancreatic parenchymal atrophy (FPPA). T2 magnetic resonance imaging shows that the pancreatic parenchyma is lacking with an irregular shape and is replaced by fat (square arrow) near the cystic lesion (arrow).

**Figure 2:**
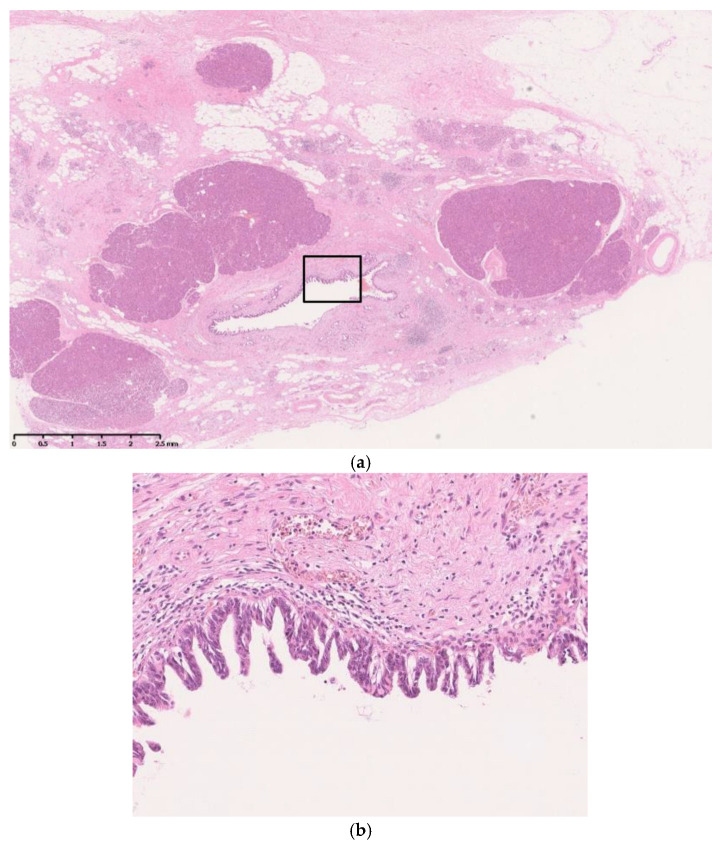
Histopathology of focal pancreatic parenchymal atrophy (FPPA). (**a**) FPPA consists of replaced adipose tissue. The margin of the area is sharp and clear, and the surrounding pancreatic parenchyma shows no significant changes, such as inflammatory cell infiltration, fibrosis, or atrophy (hematoxylin–eosin, ×40). (**b**) FPPA is observed around or adjacent to the pancreatic duct with neoplastic changes in the pancreatic ductal epithelium (square part of **a**) (hematoxylin–eosin, ×200).

**Figure 3:**
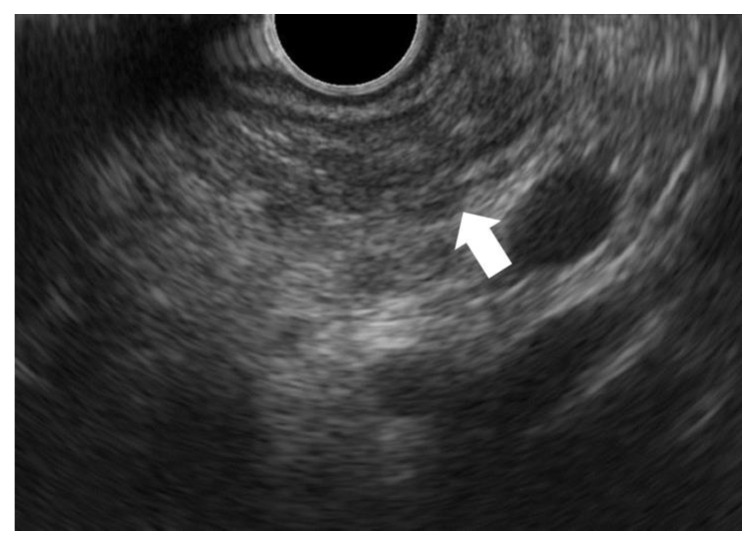
Endoscopic ultrasonography (EUS) finding of focal pancreatic parenchymal atrophy (FPPA). FPPA can be observed on EUS as a blurred hypoechoic area (arrow).

**Figure 4:**
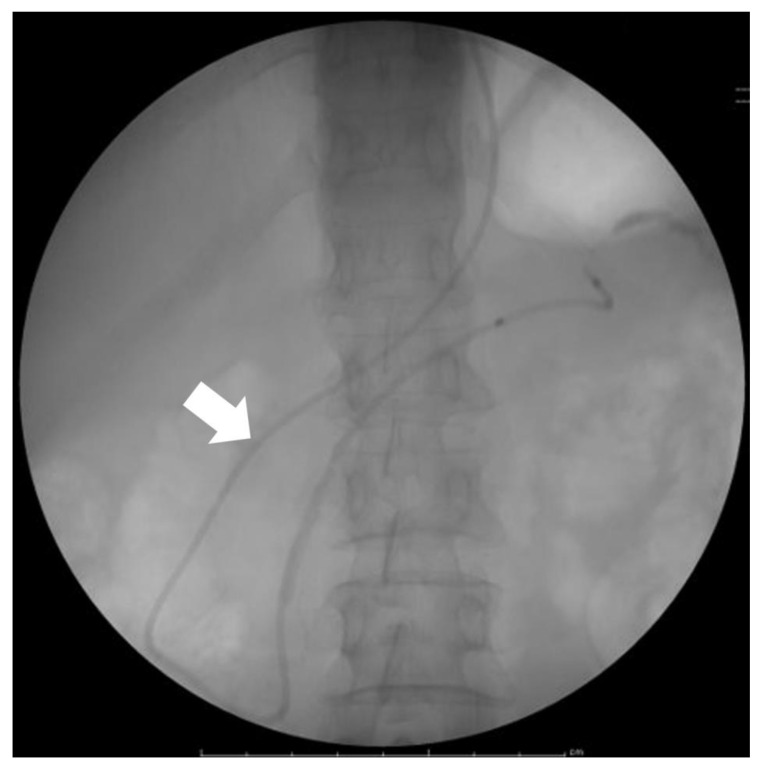
Endoscopic nasopancreatic drainage tube (arrow) placement for serial pancreatic juice aspiration cytologic examination (SPACE).

**Figure 5:**
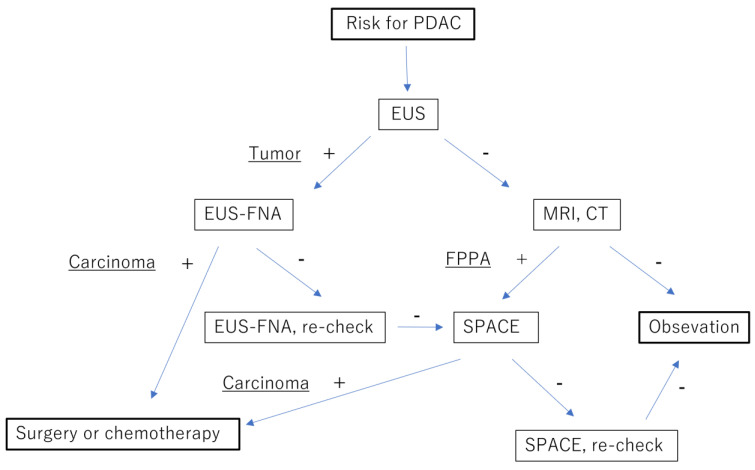
Proposal of a diagnostic strategy for pancreatic cancer at the early stage. Endoscopic ultrasound (EUS) is the first-line treatment for patients at risk of pancreatic ductal adenocarcinoma (PDAC). Endoscopic ultrasound-guided fine-needle aspiration (FNA) is performed when a tumor is identified. If a histopathological finding of PDAC is not obtained despite repeated EUS-FNA, serial pancreatic juice aspiration cytologic examination (SPACE) is undertaken. If no tumor is detected on EUS, but focal pancreatic parenchymal atrophy (FPPA) is recognized on magnetic resonance imaging (MRI) or computed tomography (CT), SPACE is selected. PDAC, pancreatic ductal adenocarcinoma; EUS, endoscopic ultrasonography; EUS-FNA, EUS-guided fine-needle aspiration; MRI, magnetic resonance imaging; CT, computed tomography; FPPA, focal pancreatic parenchymal atrophy; SPACE, serial pancreatic juice aspiration cytologic examination.

**Table 1:** Advantages and disadvantages of diagnostic imaging methods.

	Advantage	Disadvantage	Commonly Detectable Tumor Size
**US**	Easy Low cost	Poor detection of the pancreas and pancreatic lesions	>10 mm (Location limited)
**CT**	Easy Observation of the entire pancreas	Radiation exposure Poor detection of small PDACs	>20 mm
**MRI**	Easy Observation of the entire pancreas	Poor detection of small PDACs	>10 mm
**PET**	Easy Observation of the entire pancreas	Radiation exposure Poor detection of small PDACs Expensive	>10 mm
**EUS**	Detection of very small PDACs Obtaining tissue for histopathological diagnosis	Invasive Technical difficulty	>5 mm

US, ultrasonography; CT, computed tomography; MRI, magnetic resonance imaging; PET, positron emission tomography; EUS, endoscopic ultrasonography; PDAC, pancreatic ductal adenocarcinoma.

## References

[1] Ilic M., Ilic I. (2016). Epidemiology of Pancreatic Cancer. World J. Gastroenterol..

[2] Bengtsson A., Andersson R., Ansari D. (2020). The Actual 5-Year Survivors of Pancreatic Ductal Adenocarcinoma Based on Real-World Data. Sci. Rep..

[3] Lee H.S., Park S.W. (2016). Systemic Chemotherapy in Advanced Pancreatic Cancer. Gut Liver.

[4] Bockhorn M., Uzunoglu F.G., Adham M., Imrie C., Milicevic M., Sandberg A.A., Asbun H.J., Bassi C., Büchler M., Charnley R.M. (2014). Borderline Resectable Pancreatic Cancer: A Consensus Statement by the International Study Group of Pancreatic Surgery (ISGPS). Surgery.

[5] Egawa S., Toma H., Ohigashi H., Okusaka T., Nakao A., Hatori T., Maguchi H., Yanagisawa A., Tanaka M. (2012). Japan Pancreatic Cancer Registry; 30 Year Anniversary: Japan Pancreas Society. Pancreas.

[6] Patel C.M., Sahdev A., Reznek R.H. (2011). CT, MRI and PET Imaging in Peritoneal Malignancy. Cancer Imaging.

[7] Leschka S., Alkadhi H., Wildermuth S., Marincek B. (2005). Multi-detector Computed Tomography of Acute Abdomen. Eur. Radiol..

[8] Iafrate F., Ciolina M., Sammartino P., Baldassari P., Rengo M., Lucchesi P., Sibio S., Accarpio F., Di Giorgio A.D., Laghi A. (2012). Peritoneal Carcinomatosis: Imaging with 64-MDCT and 3T MRI with Diffusion-Weighted Imaging. Abdom. Imaging.

[9] Ellis M., Powell J.T., Greenhalgh R.M. (1991). Limitations of Ultrasonography in Surveillance of Small Abdominal Aortic Aneurysms. Br. J. Surg..

[10] Saunders H.M. (1991). Ultrasonography of the Pancreas. Probl. Vet. Med..

[11] Engjom T., Sangnes D.A., Havre R.F., Erchinger F., Pham K.D.C., Haldorsen I.S., Gilja O.H., Dimcevski G. (2017). Diagnostic Accuracy of Transabdominal Ultrasound in Chronic Pancreatitis. Ultrasound Med. Biol..

[12] Liu L., Huang X., Li X., Ma L., Zhao X., Yang X. (2019). Ultrasound Combined with Computed Tomography in Pancreatic Cancer. J. BUON.

[13] Kanno A., Masamune A., Hanada K., Maguchi H., Shimizu Y., Ueki T., Hasebe O., Ohtsuka T., Nakamura M., Takenaka M. (2018). Multicenter Study of Early Pancreatic Cancer in Japan. Pancreatology.

[14] Yoon S.H., Lee J.M., Cho J.Y., Lee K.B., Kim J.E., Moon S.K., Kim S.J., Baek J.H., Kim S.H., Kim S.H. (2011). Small (≤20 Mm) Pancreatic Adenocarcinoma: Analysis of Enhancement Patterns and Secondary Signs with Multiphasic Multidetector CT. Radiology.

[15] Harinck F., Konings I.C.A.W., Kluijt I., Poley J.W., van Hooft J.E., van Dullemen H.M., Nio C.Y., Krak N.C., Hermans J.J., Aalfs C.M. (2016). A Multicentre Comparative Prospective Blinded Analysis of EUS and MRI for Screening of Pancreatic Cancer in High-Risk Individuals. Gut.

[16] Robertis R.D., Martini P.T., Demozzi E., Corso F.D., Bassi C., Pederzoli P., D’Onofrio M. (2015). Diffusion-Weighted Imaging of Pancreatic Cancer. World J. Radiol..

[17] Zukotynski K., Kim C.K. (2014). Abdomen: Normal Variations and Benign Conditions Resulting in Uptake on FDG-PET/CT. PET Clin..

[18] Rijkers A.P., Valkema R., Duivenvoorden H.J., van Eijck C.H.J. (2014). Usefulness of F-18-Fluorodeoxyglucose Positron Emission Tomography to Confirm Suspected Pancreatic Cancer: A Meta-analysis. Eur. J. Surg. Oncol..

[19] Helmstaedter L., Riemann J.F. (2008). Pancreatic Cancer—EUS and Early Diagnosis. Langenbecks Arch. Surg..

[20] Yasuda I., Iwashita T., Doi S., Nakashima M., Moriwaki H. (2011). Role of EUS in the Early Detection of Small Pancreatic Cancer. Dig. Endosc..

[21] Kitano M., Yoshida T., Itonaga M., Tamura T., Hatamaru K., Yamashita Y. (2019). Impact of Endoscopic Ultrasonography on Diagnosis of Pancreatic Cancer. J. Gastroenterol..

[22] Terada S., Kikuyama M., Kawaguchi S., Kanemoto H., Yokoi Y., Kamisawa T., Kuruma S., Chiba K., Honda G., Horiguchi S. (2019). Proposal for Endoscopic Ultrasonography Classification for Small Pancreatic Cancer. Diagnostics.

[23] Debongnie J.C., Pauls C., Fievez M., Wibin E. (1981). Prospective Evaluation of the Diagnostic Accuracy of Liver Ultrasonography. Gut.

[24] Bhutani M.S., Koduru P., Joshi V., Saxena P., Suzuki R., Irisawa A., Yamao K. (2016). The Role of Endoscopic Ultrasound in Pancreatic Cancer Screening. Endosc. Ultrasound.

[25] Hewitt M.J., McPhail M.J., Possamai L., Dhar A., Vlavianos P., Monahan K.J. (2012). EUS-Guided FNA for Diagnosis of Solid Pancreatic Neoplasms: A Meta-analysis. Gastrointest. Endosc..

[26] Hébert-Magee S., Bae S., Varadarajulu S., Ramesh J., Frost A.R., Eloubeidi M.A., Eltoum I.A. (2013). The Presence of a Cytopathologist Increases the Diagnostic Accuracy of Endoscopic Ultrasound-Guided Fine Needle Aspiration Cytology for Pancreatic Adenocarcinoma: A Meta-analysis. Cytopathology.

[27] Bang J.Y., Hawes R., Varadarajulu S. (2016). A Meta-analysis Comparing ProCore and Standard Fine-Needle Aspiration Needles for Endoscopic Ultrasound-Guided Tissue Acquisition. Endoscopy.

[28] Karsenti D., Tharsis G., Zeitoun J.D., Denis P., Perrot B., Coelho J., Bellaiche G., Charbit L., Hakoune J.J., Doumet S. (2019). Comparison of 20-Gauge Procore^®^ and 22-Gauge Acquire^®^ Needles for EUS-FNB of Solid Pancreatic Masses: An Observational Study. Scand. J. Gastroenterol..

[29] Nakai Y., Isayama H., Chang K.J., Yamamoto N., Hamada T., Uchino R., Mizuno S., Miyabayashi K., Yamamoto K., Kawakubo K. (2014). Slow Pull Versus Suction in Endoscopic Ultrasound-Guided Fine-Needle Aspiration of Pancreatic Solid Masses. Dig. Dis. Sci..

[30] Bang J.Y., Magee S.H., Ramesh J., Trevino J.M., Varadarajulu S. (2013). Randomized Trial Comparing Fanning with Standard Technique for Endoscopic Ultrasound-Guided Fine-Needle Aspiration of Solid Pancreatic Mass Lesions. Endoscopy.

[31] Kitano M., Kudo M., Yamao K., Takagi T., Sakamoto H., Komaki T., Kamata K., Imai H., Chiba Y., Okada M. (2012). Characterization of Small Solid Tumors in the Pancreas: The Value of Contrast-Enhanced Harmonic Endoscopic Ultrasonography. Am. J. Gastroenterol..

[32] Cui X.W., Chang J.M., Kan Q.C., Chiorean L., Ignee A., Dietrich C.F. (2015). Endoscopic Ultrasound Elastography: Current Status and Future Perspectives. World J. Gastroenterol..

[33] Kawaguchi S., Kikuyama M., Satoh T., Terada S., Kanemoto H., Arai K. (2017). Minimally Invasive Ductal Pancreatic Carcinoma without Low Echoic Area on Endoscopic Ultrasound Examination: A Case Report. J. Jpn. Pancreas Soc..

[34] Kalady M.F., Peterson B., Baillie J., Onaitis M.W., Abdul-Wahab O.I., Howden J.K., Jowell P.S., Branch M.S., Clary B.M., Pappas T.N. (2004). Pancreatic Duct Strictures: Identifying Risk of Malignancy. Ann. Surg. Oncol..

[35] Nakahodo J., Kikuyama M., Nojiri S., Chiba K., Yoshimoto K., Kamisawa T., Horiguchi S.I., Honda G. (2020). Focal Parenchymal Atrophy of Pancreas: An Important Sign of Underlying High-Grade Pancreatic Intraepithelial Neoplasia without Invasive Carcinoma, i.e., Carcinoma in Situ. Pancreatology.

[36] Kikuyama M., Nakahodo J., Honda G., Horiguchi S., Suzuki M., Chiba K., Tabata H., Ome Y., Kamisawa T. (2021). Effectiveness of Focal Pancreatic Parenchymal Atrophy in Diagnosing High-Grade Pancreatic Intraepithelial Neoplasia/Carcinoma In Situ. MRAJ.

[37] Nakahodo J., Kikuyama M., Fukumura Y., Horiguchi S.I., Chiba K., Tabata H., Suzuki M., Kamisawa T. (2022). Focal Pancreatic Parenchyma Atrophy Is a Harbinger of Pancreatic Cancer and a Clue to the Intraductal Spreading Subtype. Pancreatology.

[38] Toshima F., Watanabe R., Inoue D., Yoneda N., Yamamoto T., Sasahira N., Sasaki T., Matsuyama M., Minehiro K., Tateishi U. (2021). CT Abnormalities of the Pancreas Associated with the Subsequent Diagnosis of Clinical Stage I Pancreatic Ductal Adenocarcinoma More Than 1 Year Later: A Case-Control Study. AJR.

[39] Kameya S., Kuno N., Kasugai T. (1981). The Diagnosis of Pancreatic Cancer by Pancreatic Juice Cytology. Acta Cytol..

[40] Iiboshi T., Hanada K., Fukuda T., Yonehara S., Sasaki T., Chayama K. (2012). Value of Cytodiagnosis Using Endoscopic Nasopancreatic Drainage for Early Diagnosis of Pancreatic Cancer: Establishing a New Method for the Early Detection of Pancreatic Carcinoma In Situ. Pancreas.

[41] Satoh T., Kikuyama M., Kawaguchi S., Kanemoto H., Muro H., Hanada K. (2017). Acute Pancreatitis-Onset Carcinoma In Situ of the Pancreas with Focal Fat Replacement Diagnosed Using Serial Pancreatic-Juice Aspiration Cytologic Examination (SPACE). Clin. J. Gastroenterol..

[42] Kikuyama M., Nakahodo J., Honda G., Suzuki M., Horiguchi S.I., Chiba K., Tabata H., Ome Y., Uemura S.I., Kawamoto Y. (2023). Pancreatic Duct Epithelial Malignancy Suggested by Large Focal Pancreatic Parenchymal Atrophy in Cystic Diseases of the Pancreas. Pancreatology.

[43] Bhatia M. (2004). Apoptosis of Pancreatic Acinar Cells in Acute Pancreatitis: Is It Good or Bad?. J. Cell. Mol. Med..

[44] Storz P. (2017). Acinar Cell Plasticity and Development of Pancreatic Ductal Adenocarcinoma. Nat. Rev. Gastroenterol. Hepatol..

[45] Izumi Y., Hanada K., Okazaki A., Minami T., Hirano N., Ikemoto J., Kanemitsu K., Nakadoi K., Shishido T., Katamura Y. (2019). Endoscopic Ultrasound Findings and Pathological Features of Pancreatic Carcinoma In Situ. EIO.

[46] Kawaguchi S., Kikuyama M., Satoh T., Terada S. (2018). Use of Nasopancreatic Drainage for Severe Post-endoscopic Retrograde Cholangiopancreatography Pancreatitis: A Case Series. Intern. Med..

[47] Kikuyama M., Kamisawa T., Kuruma S., Chiba K., Kawaguchi S., Terada S., Satoh T. (2018). Early Diagnosis to Improve the Poor Prognosis of Pancreatic Cancer. Cancers.

[48] Furukawa H., Iwata R., Moriyama N. (2001). Growth Rate of Pancreatic Adenocarcinoma: Initial Clinical Experience. Pancreas.

[49] Nishida K., Kaneko T., Yoneda M., Nakagawa S., Ishikawa T., Yamane E., Nishioka B., Miyamoto Y., Takano H., Yoshikawa T. (1999). Doubling Time of Serum CA 19–9 in the Clinical Course of Patients with Pancreatic Cancer and Its Significant Association with Prognosis. J. Surg. Oncol..

